# Examining Changes in Compassion Satisfaction, Burnout, Secondary Traumatic Stress and Work-Related Quality-of-Life of Rural Surgical and Obstetrical Nurses in British Columbia During the COVID-19 Pandemic

**DOI:** 10.1177/08445621241305194

**Published:** 2024-12-19

**Authors:** Kathrin Stoll, Jenna Treissman, Gal Av-Gay, Jude Kornelsen

**Affiliations:** 1 Department of Family Practice, Center for Rural Health Research, Vancouver, Canada; 2 Department of Family Practice, 512469 University of British Columbia, Vancouver, Canada; 3 Faculty of Medicine, 512469 University of British Columbia, Vancouver, Canada

**Keywords:** COVID-19, pandemic, rural, surgical, obstetrical, nursing, quality-of-life, rural surgical and obstetrical networks, RSON

## Abstract

**Background:**

This survey of rural nurses’ experiences is part of a program evaluation of the Rural Surgical and Obstetrical Networks (RSON), a five-year initiative (2018–2023) to strengthen and stabilize rural hospitals in British Columbia (BC), Canada.

**Purpose:**

Our aim was to measure changes in professional and work-related quality-of-life of rural surgical and obstetrical nurses in eight communities across BC and determine if the RSON initiative mitigated impacts of the COVID-19 pandemic on nurses’ quality of life.

**Methods:**

This longitudinal evaluation was administered via online surveys in 2021 and 2023. Work-related quality of life was measured with 23 items that assess job satisfaction, general wellbeing, work-life balance, stress level experienced at work, control, and working conditions. Professional quality of life was measured along three dimensions: compassion satisfaction (CS), burnout, and secondary traumatic stress (STS) (10 items each). Responses were linked by code and changes in quality of life were analyzed using paired Student's t-test.

**Results:**

107 nurses participated at time 1 and 28 at time 2. Burnout and secondary traumatic stress scores at time 1 were lower among older nurses and those with children. Over the two-year period (2021 to 2023), significant increases were observed in burnout (*p* < 0.001), and secondary traumatic stress (*p* = 0.04), while work-related Quality-of-Life decreased significantly (*p* = 0.04). Compassion satisfaction decreased over time, though not statistically significant.

**Conclusions:**

While the RSON initiative could not mitigate decreases in professional and work-related quality-of-life during COVID-19, it offered opportunities for clinical education and professional development among rural nurses.

## Background & purpose

Nurses are an essential part of surgical and perinatal care teams in rural areas of Canada. They share a common set of characteristics, including the need to be versatile and prepared, having multiple skills and an expanded scope of practice, caring for a higher proportion of Indigenous patients and the blurring of professional boundaries as many of their patients are known to them (Pavloff et al., 2022).

A national survey in 2014/2015 with 3822 regulated nurses who practiced in rural and remote areas of Canada found that the most commonly cited reasons attracting nurses to rural communities were the location of the community (56%), interest in the practice setting (53%) and income (45%) (MacLeod et al., 2017). The same three factors were reported when nurses were asked about why they continue to work in rural communities, although income was a stronger retention than recruitment incentive. Two-thirds of surveyed nurses grew up in a community with less than 10,000 people, highlighting the connection between personal history and choice of practice location (MacLeod et al., 2017).

Rural nurses face many challenges associated with their practice setting including nursing and physician shortages, high workloads with limited resources and difficulty retaining specialized skills that are used infrequently (Ross & Bell, 2009). They express discomfort around the lack of anonymity in their community and the awareness that their actions both inside and outside of the work environment may impact their reputations as nurses (Mills et al., 2007).

Interviews with 30 nurses at four hospitals in and around Cranbrook (BC) shed light on the working conditions of nurses and their experiences with providing maternity care. Participants noted that a large part of their daily work was focused on ‘anticipating problems and protecting patients’ (p.122). Other themes that emerged from the interviews included being prepared for emergencies, careful watching for early signs that something might be wrong, protecting patients by mobilizing additional resources, advocating for patient safety and taking actions in emergency situations. Findings revealed the complexity of rural nursing practice, nurses’ commitment to patient safety and their difficulties in ensuring adequate nursing coverage. Arranging for maternal and/or neonatal transport was especially stressful and time intensive for nurses and physicians (MacKinnon, 2011).

Despite these challenges, nurses also describe many benefits of working in small communities such as the ability to develop close relationships with patients and colleagues, the diversity of skills applied to clinical problems, and the ability to care for one's own community members (Ross & Bell, 2009). Each of these positive and negative elements of working in a rural community contribute to the quality-of-life (QoL) of rural and remote nurses.

The impact of the COVID-19 pandemic on nurses in Canada has been profound: more than 9 of 10 nurses (92%) reported that they experienced greater work stress in the fall of 2021 than before the pandemic and 83.7% reported that their workload was higher than before the pandemic (Statistics Canada, 2022).

When asked about their mental health status compared with before the COVID-19 pandemic, nurses were most likely to respond that their mental health was somewhat or much worse now (52.4% of nurses versus 46.4% of physicians and 44.1% of other healthcare workers). Many nurses were planning to leave the profession for reasons other than retirement, especially those with 5 years or less work experience (Statistics Canada, 2022).

No studies were identified that assessed professional and/or work-related quality of life among rural nurses in BC, using validated scales. Some studies reported on related constructs such as job satisfaction. For example, an analysis of predictors of job satisfaction among rural and remote acute care nurses in Canada (*n* = 944) showed that 33% of the variation in job satisfaction scores could be predicted by four interrelated factors: available and up-to-date equipment and supplies, satisfaction with scheduling and shifts, lower psychological job demands, and home community satisfaction (Penz et al., 2008).

The current analysis of professional and work-related quality of life of rural nurses is part of a program evaluation of a 5-year initiative aimed at improving quality of care at rural hospitals in British Columbia (Canada). (Kornelsen et al., 2024a, in press)

### Closure of maternity services in rural BC

Over the past 20 years, approximately one-third of hospitals in British Columbia (BC) (Canada), particularly those in rural and low volume settings, have ceased to provide maternity services (Hutcheon et al., 2017). Closure of local maternity services in rural areas carries with it a host of negative consequences for patients and their health care providers (Grzybowski et al., 2007, 2011; Kornelsen et al., 2011). In one study with nurses, physicians & hospital administrators (*n* = 30) from 4 rural communities in British Columbia, nurses and physicians noted difficulties in maintaining both obstetric and general skills in low volume settings. While physicians had opportunities to travel to larger centers to keep up their skills, nurses lacked the personal and professional resources to leave their communities to travel to higher volume centers. (Grzybowski et al., 2007)

### The rural surgical and obstetrical networks (RSON) of BC

The Rural Surgical and Obstetrical Networks (RSON), which was funded by the Joint Standing Committee on Rural Issues (a collaborative committee between BC's Ministry of Health and the professional physician's association, Doctors of BC) is a 5 year initiative (2018 and 2023) designed to support and sustain low-volume surgical services in rural BC. RSON was implemented, in the hopes of stabilizing rural health services and ensuring the availability of perinatal and surgical care ‘close to home’ for rural and remote patients in eight communities across the province. RSON-supported hospitals offered several procedures for low acuity patients, including orthopedic surgeries, hernia repair, appendectomies, gynecological procedures, Cesarean sections and endoscopies (Av-Gay et al., 2024).

RSON provided rural hospitals in British Columbia (Canada) with funding to increase the scope and volume of surgical procedures that the hospitals can offer, engage hospital teams in continuous quality improvement projects, and enable clinical coaching relationships between health care providers at rural and referral hospitals (Kornelsen et al., 2024a).

The evaluation team assessed the impact of RSON funding by analyzing (1) outcomes of patients using health administrative data, (2) patient experiences with surgical care via phone interviews 30 days post-surgery, (3) surveys with health care providers and (4) annual interviews and focus groups with 169 healthcare providers and administrators (including 56 registered nurses and 4 nurse educators) to understand whether the RSON initiative made a difference in strengthening and stabilizing rural hospital services.

Several papers have been published about the evaluation of the RSON initiative. Analyses of perinatal and surgical outcomes at RSON supported hospitals compared to referral sites showed lower rates of adverse maternal and newborn outcomes and lower rates of surgical complications at RSON supported hospitals, after controlling for patient characteristics (Kornelsen et al., 2024a, in press; Stoll et al., 2024). In addition, the evaluation team published qualitative analyses of how the RSON initiative stabilized local maternity services, how it improved team function, and relationships between rural providers and colleagues at regional referral sites (Kornelsen et al., 2023a; Kornelsen et al., 2023b; Kornelsen et al., 2023c). Additional papers describe the nature and impact of CQI activities at RSON supported hospitals (Kornelsen et al., 2024b), and the views of outreach specialists who served RSON hospitals (Parajulee et al., 2024). These papers demonstrate the positive impact of RSON funding as well as barriers encountered along the way.

In the first two years of RSON, nurses reported high levels of burnout and work dissatisfaction during interviews with the evaluation team, which led the team to design a purposeful survey to better understand these issues. The implementation of strategies to reduce burnout and improve working conditions for rural nurses relies on a thorough understanding of the issue and is a critical component of the effort to stabilize and sustain safe and accessible health services for rural patients and their families.

The goal of the analysis was to better understand the experiences of two specialty groups of rural nurses (surgical and obstetric) during the COVID 19 pandemic and to determine whether the RSON initiative had a positive influence on nurses’ professional and work-related Quality of Life (QoL).

Specifically, we:

1. Examined associations between (1) compassion satisfaction, burnout, secondary traumatic stress (professional QoL), and (2) work-related QoL scores at time 1 (2021) and socio-demographic characteristics.
2. Evaluated differences in compassion satisfaction, burnout, secondary traumatic stress (professional QoL) and work-related QoL at time 1 (2021) and time 2 (2023).

## Methods and procedures

For this longitudinal evaluation we recruited surgical and obstetric nurses who practised in eight RSON-supported rural hospitals in British Columbia. The first hospital began participating in the RSON initiative in April 2018 and the last hospital in September 2020. An online survey was administered to nurses at two time points: mid-intervention (time 1: January/February/March 2021) and post-intervention (time 2: January/February 2023). Both surveys included validated scales measuring professional and work related QoL and survey 1 also included 9 socio-demographic questions and items about nurses experiences during the COVId 19 pandemic. The surveys were mounted on the Canadian platform Qualtrics and a link and QR code to the online survey were embedded in the recruitment materials. Surgical and obstetrical nurses over the age of 19 who worked at one of the RSON supported hospitals were eligible to participate.

## Measures

The Professional Quality-of-Life Scale (ProQoL-5) is the most commonly used measure of both the positive and negative effects of working with people (Stamm, 2010) and is defined as “the quality one feels in relation to their work as a helper” (Stamm, 2010). The ProQoL-5 incorporates 30 items across three domains with 10 items in each subdomain: compassion satisfaction (CS), burnout, and secondary traumatic stress (STS) (Stamm, 2010). CS measures the joy nurses derive from doing their work, helping patients and feeling satisfied with their contributions at work. Higher scores indicate higher CS. Burnout is characterized as feelings of exhaustion and frustration in one's work, the sense that one's efforts do not make a difference, or feelings of hopelessness and futility (Stamm, 2010). Higher scores mean higher levels of burnout. STS is defined as work-related exposure to people who have previously, or who are currently experiencing stressful or traumatic events (Stamm, 2010). STS include symptoms similar to those experienced by individuals suffering from post-traumatic stress disorder (PTSD). For example, nurses who experience STS may suffer sleep difficulties and intrusive images, or they may alter their behaviour to avoid reminders of their traumatic experience (Stamm, 2010). Higher scores indicate more severe STS. Respondents were asked to read several statements and respond based on how frequently they have experienced these feelings in the last 30 days. Responses range from 1 (never) to 5 (very often) (Stamm, 2010). Scores were calculated for each of the three subdomains. In the current sample, the internal consistency reliability of the three subscales was high: 0.91 for the CS scale, 0.82 for the burnout scale and 0.85 for the STS scale.

The Work-Related Quality-of-Life (WRQoL) scale is a 23-item scale designed specifically for healthcare professionals to capture six factors related to their quality of work and personal life (Van Laar et al., 2007). These include job satisfaction, general wellbeing, work-life balance, stress level experienced at work, control, and working conditions. For example, under the domain ‘job and career satisfaction’, healthcare professionals are asked to respond to the statement “I am satisfied with the career opportunities available to me” (Laar et al., 2007). Similarly, under the domain of ‘home-work interface’, they are asked to respond to the statement “My current working hours and patterns suit my personal circumstances” (Laar et al., 2007). The authors of this scale suggest that stressors, pressure and poor working conditions contribute to ill health among healthcare professionals, which impacts the quality of patient care they are able to deliver (Laar et al., 2007). Possible responses range from 1 (strongly disagree) to 5 (strongly agree) with higher scores indicating higher work related QoL. The WRQoL items had a Cronbach's alpha of 0.90 in the current study, indicating high internal consistency reliability of the scale among rural nurses. Subscale alphas ranged from 0.67–0.89 (see Table 1). Because of lower alpha values for some of the subscales, we decided to proceed with an analysis of the overall scale score.

**Table 1.** Summary of Scales – Time 1 (2021).

	Number of items	n	Range of scores	Median (5^th^, 95^th^ percentile)	Cronbach's alpha
Compassion satisfaction	10	107	16–50	39.0 (29.4, 47.6)	0.91
Burnout	10	106	10–39	24.0 (15.0, 33.0)	0.82
Secondary traumatic stress	10	107	12–42	23.0 (14.0, 33.6)	0.85
Work related Quality of Life	23	106	1.9–4.8	3.5 (2.5, 4.4)	0.90
General Well Being (GWB)	6	107	1.7–5.0	4.0 (2.5, 4.8)	0.89
Home-Work Interface (HWI)	3	106	1–5	3.3 (1.7,4.7)	0.74
Job Career Satisfaction (JCS)	6	107	1.8–5	3.7 (2.5,4.5)	0.72
Control at Work (CAW)	3	107	1.3–5.0	3.3 (1.3–5.0)	0.67
Working Conditions (WCS)	3	107	1.3–5.0	3.7 (2.1–4.5)	0.72
Stress at Work (SAW)	2	107	1–5	3.0 (1.5,4.5)	0.76

At time 1 we also included nine items that measure how rural nurses were affected by the COVID-19 pandemic. Each item had 5 response options, ranging from 1 (never) to 5 (very often). Items included “The COVID-19 pandemic has had a significantly negative impact on the quality of my working life” and “Due to my work as a healthcare provider during the COVID-19-19 pandemic, I fear for the health and wellbeing of my family.”

## Recruitment and data collection

At time 1 (2021) RSON project coordinators and nurse managers recruited nurses via e-mail and recruitment flyers that were posted in maternity and surgical units at the RSON supported hospitals. They could complete the survey on their mobile phones or computers at a time and place that was convenient to them. Nurses who participated in the baseline survey at time 1 received a $40 gift card. The 85 nurses who completed the baseline survey and agreed to be recontacted received an invitation in January 2023, to participate in the second survey. Responses were linked via a unique ID number.

## Quantitative analysis

We reported median scores and 5^th^ and 95^th^ percentiles for each of the scales, stratified by socio-demographic characteristics of respondents. At times, response options were collapsed to ensure cell counts were robust and to avoid inadvertent participant identification. We used paired samples t-test to compare mean scale scores at time 1 and time 2 and Spearman's rho correlation coefficient, to understand how scale scores were associated with each other.

## Results

Between January 6 and March 7, 2021, 146 rural surgical and obstetrical nurses from the eight RSON communities responded to the online questionnaire and between 104 and 107 completed the QoL scales (see Table 1) (response rate: time 1:107/159 = 67%; time 2: 28/85 = 33%). Most participants identified as female (93%) and the average age of respondents was 41 years. The majority of respondents (66%) were married, while 34% were never married or divorced. Most participants reported having one or more children (66.0%). Close to 40% of nurses had worked in the RSON community for more than 10 years.

### Professional & work-related QoL at time 1

At time 1 (early 2021), the median level of compassion satisfaction experienced by RSON nurses was 39.0, which represents a moderate-high level of satisfaction with one's work in a helping profession. The median burnout score for RSON nurses was 24.0, which represents a low-average level of burnout. Finally, the median secondary traumatic stress (STS) score was 23.0 which translates to a low-average level of STS (Stamm). Nurses at RSON supported hospitals reported a median WRQoL score of 3.5, meaning that on average they rated their work-related quality of life between neutral and good.

Scores for CS, burnout, STS, and WRQoL were largely consistent across socio-demographic groups, with some exceptions. Burnout and STS scores were lower among nurses aged 40 or older. STS scores were also lower among nurses who had 10 or more years of practise experience. Married nurses reported higher burnout, and more secondary traumatic stress compared to their unmarried counterparts. However, having children (of any age) was associated with higher QoL. (see Table 2).

**Table 2.** Median Scale Scores (with 5^th^ and 95^th^ Percentiles), Stratified by Socio-demographic Characteristics – Time 1 (2021).

	n (%)	Compassion Satisfaction	Burnout	Secondary traumatic stress	Work-related Quality of Life
Age					
39 or younger	43 (42.2)	39.0 (25.6–45.0)	24.0 (15.4–35.2)	24.0 (13.6–35.4)	3.5 (2.8–4.5)
40 or older	59 (57.8)	39.0 (33.0–48.0)	23.0 (15.0–33.1)	23.0 (14.0–34.0)	3.5 (2.1–4.4)
Years in practice					
0–10 years	34 (33.0)	39.0 (22.8–42.3)	24.0 (16.3–36.8)	24.0 (16.8–33.8)	3.5 (2.9–4.3)
Over 10 years	69 (67.0)	39.0 (31.5–49.0)	24.0 (14.5–33.0)	23.0 (13.0–34.0)	3.5 (2.2–4.5)
Years in current community					
0–10 years	63 (61.2)	39.0 (29.4–49.6)	24.0 (14.0–32.0)	22.0 (14.2–32.8)	3.5 (2.4–4.5)
Over 10 years	40 (38.8)	39.5 (28.1–45.0)	24.0 (17.0–34.0)	23.0 (13.1–37.8)	3.5 (2.5–4.2)
Household income					
Under 120 k	44 (42.7)	39.0 (30.3–49.0)	24.0 (14.0–31.8)	23.0 (12.3–31.8)	3.5 (2.5–4.7)
120 k or more	59 (57.3)	39.0 (28.0–45.0)	23.5 (16.9–34.1)	23.0 (15.0–36.0)	3.5 (2.1–4.3)
Children					
No	35 (34.0)	38.0 (22.4–45.0)	25.0 (17.8–36.6)	25.0 (17.0–34.4)	3.4 (2.7–4.3)
Yes	68 (66.0)	39.0 (33.0–49.1)	23.0 (14.0–32.0)	22.0 (13.0–32.0)	3.6 (2.4–4.5)
Children under 6					
No	82 (79.6)	39.0 (28.2–45.9)	24.0 (16.1–33.0)	23.0 (14.0–33.0)	3.5 (2.5–4.4)
Yes	21 (20.4)	39.0 (33.2–49.6)	24.0 (10.4–36.5)	23.0 (12.4–41.6)	3.6 (2.0–4.8)
Marital status					
Legally married	67 (65.7)	39.0 (28.4–47.2)	24.0 (14.0–33.6)	23.0 (13.8–34.0)	3.5 (2.5–4.3)
Single	35 (34.3)	39.0 (29.6–46.8)	23.0 (15.8–33.8)	22.0 (13.8–33.6)	3.5 (2.4–4.5)

### Associations between scale scores

All scale scores were significantly correlated with each other (*p* < 0.001) (see Table 3), indicating strong conceptual overlap between the scales. The strongest positive relationship was observed with secondary traumatic stress and burnout.

**Table 3.** Correlations Between Scale Scores – Time 1 (2021).

	Work related Quality of Life	Compassion satisfaction	Burnout	Secondary traumatic stress
Work related Quality of Life	1.00	0.62**	−0.69**	−0.54**
Compassion satisfaction	0.62**	1.00	−0.63**	−0.29**
Burnout	−0.69**	−0.63**	1.00	0.64**
Secondary traumatic stress	−0.54**	−0.29**	0.64**	1.00

**p < 0.001

### Stress associated with COVID 19 at time 1 (2021)

In early 2021, one in two nurses (51.9%) reported that the COVID-19 pandemic has had a significant negative impact on the quality of their working life; and 41.3% noted that the pandemic significantly increased the amount of stress and anxiety they felt. In comparison, less than one in three nurses (27.9%) said that the pandemic has had a significant negative impact on their ability to do their job well (see Table 4).

**Table 4.** Nurses’ Attitudes Towards the COVID-19 Pandemic (*n* = 104) – Time 1 (2021).

	n (%) who agreed or strongly agreed
The COVID-19 pandemic has had a significant negative impact on my general quality-of-life.	61 (59.6)
The COVID-19 pandemic has had a significant negative impact on the quality of my working life.	54 (51.9)
The COVID-19 pandemic has had a significant negative impact on my ability to do my job well.	29 (27.9)
Due to my work as a healthcare provider during the COVID-19 pandemic, I fear for my own health and wellbeing.	49 (47.1)
Due to my work as a healthcare provider during the COVID-19 pandemic, I fear for the health and wellbeing of my family.	60 (57.7)
Due to my work as a healthcare provider during the COVID-19 pandemic, I fear that I may transmit the virus to my patients.	32 (30.8)
I have a significantly increased amount of stress and anxiety as a result of the COVID-19 pandemic.	43 (41.3)

### Changes in quality of life over time

Of the 85 nurses who agreed to be recontacted at time 1, 28 participated at time 2. Nurses reported significantly higher burnout in 2023 compared to two years earlier (24.0 at time 1, compared to 27.7 at time 2, *t* = −3.96; df = 27, two-sided *p* < 0.001, Cohen's d effect size: −0.75). Scores on the secondary traumatic stress scale also increased significantly between 2021–2023 (23.3 at time 1 versus 25.0 at time 2, *t* = −2.13; df = 27, two-sided *p* = 0.04, Cohen's d effect size: −0.40). Compassion satisfaction decreased over time, but this decrease was not statistically significant (37.7 at time 1 versus 36.0 at time 2, *t* = 1.95; df = 27, two-sided *p* = 0.06, Cohen's d effect size: 0.37). Work related Quality of Life decreased significantly, from 3.4 in 2021 to 3.2 in 2023 (*t* = 2.12; df = 27, two-sided *p* = 0.04, Cohen's d effect size: 0.40).

### Narrative responses

Open-ended comments provided by nurses at time 2 help contextualize the quantitative findings. Three nurses specifically mentioned the RSON initiative. One nurse described the impact of staff shortages on their mental health, barriers to obtaining additional education, discomfort when working alone, lack of support from health authority leaders and the exodus of nurses because of these and other factors. The nurse appreciated the education for maternity nurses that was provided by RSON.

As we have a worsening shortage of nurses at work, I feel more anxious and less supported. I am often guilted into coming into work. The education I received for another specialty was done poorly, and I felt very taken advantage of while pursuing the education as I had to use shift trades and find nurses to cover my shifts in order to receive the education I needed. I have repeatedly asked to not work alone in a specialty area, but I am often the only specialty nurse in either the emergency department or the maternity department in our facility. More and more nurses are leaving our hospital, and I’m not sure how long I’ll be able to work in the conditions we are placed in if staffing levels don’t change soon. I have appreciated all the education from RSON that we have received and I feel like I have grown in my maternity practice from it, but the lack of support from [the health authority] and management is starting to take away the joy that I have in my job as a nurse.

Two other nurses mentioned the positive impact of RSON on skill development and competence, with one nurse pointing out that they could not attend most of the education events because of shift work.

RSON has been instrumental with keeping up on education to continue to be competent at my job. Without RSON I am worried this will change.

RSON has brought a positive impact to my workplace by increasing opportunities to participate in new learning opportunities. However, the majority of these opportunities have fallen when I work nights so I haven’t had a lot of opportunity to participate. Without RSON the level of new learning opportunities would not have been possible.

## Discussion

We found significant decreases in work-related quality-of-life and significant increases in burnout and secondary traumatic stress between 2021 and 2023 for rural obstetrical and surgical nurses. It appears that the benefits of RSON could not mitigate the effects of the escalating health care crisis unfolding in rural BC during COVID-19. There was an outflux of maternity nurses at RSON supported hospitals who left their positions for less stressful jobs in the community (Kornelsen et al., 2023c), resulting in critical nurse shortages that affected all hospitals that participated in the RSON initiative. In addition to nurses leaving hospital-based work, there was general attrition of nurses as indicated by the 24% increase in job vacancies for registered nurses and registered psychiatric nurses in Canada between the first quarter of 2022 and the first quarter of 2023 (Statistics Canada, 2023).

While the RSON initiative facilitated a shared and renewed focus on patient safety due to CQI funding and enabled one nurse at each site to take on a part time CQI role, it proved difficult to get CQI projects off the ground in the face of severe staff shortages (Kornelsen et al., 2024b). CQI nurses were often re-deployed to provide patient care and reported internal conflict when they worked on CQI projects because they were not contributing to clinical care. Recruiting additional nurses was difficult in many communities for various reasons, including the escalating cost of living and lack of housing in many of the RSON supported communities. It is not surprising that burnout and secondary traumatic stress increased significantly over the 2-year period while health systems were buckling under the pressure inflicted by the pandemic. A focus group with 11 nurses in January/February 2021 (i.e., same as time 1 data collection in the current study) showed that nurses in British Columbia were often forced to return to work after a traumatic work incident because of staff shortages and/or feelings of guilt (Oveisi et al., 2021). The inability to recover after stressful events combined with the pressure of trying to provide high quality patient care in a stressful work environment that was characterized by increased workloads, exclusion from decision making, and difficulties adhering to changing COVID protocols contributed to burnout and poor mental health among the nurses that were interviewed (Oveisi et al.,2021). In the current study compassion satisfaction did not change significantly over the two-year period, indicating that rural nurses were able to derive satisfaction from helping others through their work and felt positively about their contributions at work. These finding are supported by other studies.

Despite nurses’ concerns about the impact on their long-term mental health, participants showed strong commitment to provide high quality patient care (Oveisi et al., 2021). Similarly, in a confidential survey with 136 registered midwives from British Columbia (pre-Covid) who completed the Copenhagen Burnout Inventory, client related burnout was very low compared to personal and work-related burnout (Stoll & Gallagher, 2019). These findings in conjunction with results from the current study provide evidence that nurses and midwives derive satisfaction and joy from caring for patients, even if they experience a high level of burnout in other domains. It is also possible that they are less likely to admit negative feelings towards patients or impairments to their ability to provide high quality patient care.

### Burnout among Canadian nurses during the pandemic

A representative survey of 4467 practising nurses that was conducted in November and December 2021, on behalf of the Canadian Federation of Nurses Unions (2022) found that two in three nurses worked at least three of their last five shifts without full regular core staff; and 40% worked all 5 shifts without full regular core staff. Most nurses (85%) reported that there was not sufficient and appropriate staffing to meet the needs of patients and 2 in 3 said that the quality of care has declined in the past year. Two in three nurses reported that their mental health had declined in the past year, and 45% reported severe burnout. Burnout and fatigue were the most commonly cited reasons for wanting to leave the profession.

In a study with 425 critical care nurses who were surveyed in May/June 2021, all reported moderate to high burnout (as measured with the ProQoL scale). Qualitative comments from 147 of the nurses revealed three main sources of burnout and poor mental health: failures of leadership, traumatic experiences at work and a sense of defeat and disillusionment (Crowe et al., 2022).

In 2021 and 2023, rural nurses in BC who worked at RSON-supported hospitals reported lower levels of burnout compared to those in the national sample (Canadian Federation of Nurses Unions, 2022and compared to critical care nurses (Crowe et al., 2022). This might be due to lower rates of COVID-19 infection in rural compared to urban areas of the country, improvements to working conditions due to RSON funding or other unmeasured factors. Qualitative comments from the current study suggest that RSON funding contributed to skill development and maintenance of nurses and other analyses of the impact of the RSON initiative have documented improvements in team function (Kornelsen et al., 2023b) because nurses had more opportunities to learn alongside nursing, midwifery and physician colleagues, and were able to participate in CQI activities together (Kornelsen et al., 2023b).

Rural nurses with children reported noticeably lower burnout and secondary traumatic stress scores and higher work-related quality of life scores compared to nurses without children at time 1. The age of the child (ren) did not appear to make a difference. Being single (rather than married) and over the age of 40 was linked to lower burnout and secondary traumatic stress (STS) scores. A scoping review of factors associated with burnout among midwives showed inconclusive results with respect to the role of marital status and parenthood. Two studies found a significant association between being married and higher burnout and two other studies found that single midwives were more prone to burnout. Three studies found that being childless was linked to significantly higher burnout scores whereas two studies revealed that presence of children or the number of children in the home was significantly associated with burnout (Sidhu et al., 2020). The finding that older nurses reported lower burnout scores is not surprising. It is well documented that younger health care professionals are more vulnerable to burnout than their older colleagues (Sidhu et al., 2020; Stelnicki et al., 2021). In one study with 3257 Canadian nurses who submitted data between May-September 2019, nurses 50 and older reported lower burnout than their younger peers and those with fewer years in practise (5–10 years versus 20 or more) reported more severe burnout. (Stelnicki et al., 2021). In the current study nurses who had been in practise longer (more than 10 years) noted reduced STS, likely because they have developed coping mechanisms over time.

### Strategies to improve work related quality of life

A review of 66 Canadian and US studies about quality of work life of nurses resulted in the following recommendations: improve working conditions and supports for new graduates, offer opportunities for continuing education, promote positive relationships with colleagues, implement stress-reduction programs, and increase financial rewards (Nowrouzi et al., 2016). The RSON funding directly supported two of these strategies (opportunities for continuing education and promoting positive relationships with colleagues). Future initiative to stabilize rural health services would benefit from including tailored supports for new graduates and early career nurses (and physicians/midwives), time off to attend continuing education, assistance with finding housing for new hires, stress reduction resources (paired with time off from work to access resources) and support with utilizing financial incentives (including student loan forgiveness and claims for work-related injuries).

## Limitations

Because the study was restricted to rural nurses who worked at hospitals who participated in the RSON program, the results are not generalizable to the population of nurses in the province. The small sample size prevented us from generating more nuanced socio-demographic categories. New graduates experience unique challenges when they transition into practise, especially during COVID 19 (McMillan et al., 2023) and in the future this group should be isolated for analysis.

Only 28 nurses of 85 participated in the follow up survey, possibly because no monetary incentive was provided at time 2, some nurses had left the profession by then or did not have time or interest to participate again. The low participation at time 2 affects the validity of results as nurses who participated at time 2 might have had different experiences from those who did not participate, introducing non-response bias.

Although the Professional Quality of Life measure was originally developed for therapists, it has been widely applied to other groups, including health care professionals (Stamm, 2010) and showed high internal consistency reliability with rural nurses in the current study.

Finally, the need for the current analysis emerged during the first two years of the RSON initiative. This deviation from the traditional pre-post intervention design means that the assessment at time 1 was not done prior to the intervention but midway. We do not know levels of burnout, secondary traumatic stress, compassion satisfaction and work-related quality of life prior to the infusion of RSON funding.

## Conclusions

This paper examined three pressing issues in health services planning: the effect of the COVID-19 pandemic on nursing, the challenges of low-volume practice in rural settings, and the efficacy of system interventions to support rural nurses, in this case through BC's Rural Surgical and Obstetrical Networks initiative. The importance of understanding and responding to nurses’ experiences given this confluence is clear: low professional quality of life and high burnout impede nurses’ ability to provide quality care and can lead to high turnover rates (Canadian Federation of Nurses Unions, 2022). This, in turn, can lead to increased costs and health service destabilization (Bae, 2022). Conversely, increased work-related quality of life and job satisfaction lead to increased productivity and efficiency, resulting in optimal health system performance. Furthermore, monitoring and understanding the experiences of nurses during the COVID-19 pandemic and otherwise, allows us to identify areas for meaningful quality improvement and our actualization of a learning health system. Ultimately, however, optimizing nurses’ working environment aligns with the ethical imperative to provide a supportive, highly functional health care system.

Conversely, if the lived and living experiences of rural nurses are not well understood, the likelihood of health service destabilization noted above is increased. This includes, but is not limited to, higher rates of turnover, increased challenges attracting nurses to rural areas and the attendant use of out-sourced agency nurse to meet the health human resource needs of discrete communities. If unabated, this can lead to decreased community trust and the consequent reluctance to seek care. This broader social impact emphasizes the urgency of co-developing, with rural nurses, solutions to improve working conditions, provide clinical and social support and create other interventions to mitigate low professional and work-related quality of life among nurses.
